# Digital twin for healthcare systems

**DOI:** 10.3389/fdgth.2023.1253050

**Published:** 2023-09-07

**Authors:** Alexandre Vallée

**Affiliations:** Department of Epidemiology and Public Health, Foch Hospital, Suresnes, France

**Keywords:** healthcare, patient, digital health, digital twin, patient safety, innovation, prediction

## Abstract

Digital twin technology is revolutionizing healthcare systems by leveraging real-time data integration, advanced analytics, and virtual simulations to enhance patient care, enable predictive analytics, optimize clinical operations, and facilitate training and simulation. With the ability to gather and analyze a wealth of patient data from various sources, digital twins can offer personalized treatment plans based on individual characteristics, medical history, and real-time physiological data. Predictive analytics and preventive interventions are made possible by machine learning algorithms, allowing for early detection of health risks and proactive interventions. Digital twins can optimize clinical operations by analyzing workflows and resource allocation, leading to streamlined processes and improved patient care. Moreover, digital twins can provide a safe and realistic environment for healthcare professionals to enhance their skills and practice complex procedures. The implementation of digital twin technology in healthcare has the potential to significantly improve patient outcomes, enhance patient safety, and drive innovation in the healthcare industry.

## Introduction

Digital twin technology is emerging as a transformative force in healthcare systems, revolutionizing the way patient care is delivered. By leveraging real-time data integration, advanced analytics, and virtual simulations, digital twins offer enhanced patient care, predictive analytics, optimization of clinical operations, and training and simulation opportunities (1).

In terms of enhanced patient care, digital twins enable healthcare providers to gather and analyze a wealth of patient data from various sources, including electronic health records (EHRs), wearables, and medical devices (2). This holistic view of the patient allows for personalized treatment plans, considering individual characteristics, medical history, and real-time physiological data. With digital twins, healthcare professionals can make accurate diagnoses, monitor patients in real-time, and empower patients to actively participate in their own care (3, 4).

Predictive analytics and preventive interventions are facilitated through digital twin technology by analyzing patient data and employing machine learning algorithms. Digital twins can predict disease progression, identify high-risk individuals, and recommend preventive measures. This proactive approach improves patient safety, long-term outcomes, and resource allocation within healthcare systems (5). Thus, digital twin technology may have the potential to transform healthcare systems by leveraging real-time data integration, advanced analytics, and virtual simulations. It can enhance patient care, enables predictive analytics, optimizes clinical operations, and supports training and simulation. This review focused on the potential of digital twins in healthcare systems to improve patient outcomes, operational efficiency, and overall healthcare excellence.

### Search strategy

PubMed Medline Web of Science, Google Scholar, Scopus databases were used for the research, with only articles in English language, using the following terms: “Digital twin”, “Digital health” and “Healthcare”. Articles included in this review were both, original research, reviews, viewpoints, and opinions articles. Literature was searched from inception to 2023.

### Digital twin

In recent times, the notion of digital twin has garnered escalating attention from both researchers and engineers. As research in the field of digital twin advances, conducted by both industry and academia, the distinctions between digital twin and other related concepts have begun to fade. Initially, the scope of digital twin encompassed physical and virtual products along with their interconnections (6). This concept has evolved due to the rapid advancements in communication technology, sensor technology, big data analysis, the Internet of Things (IoT), and simulation technology (7). This growth has fueled substantial research into digital twins.

Subsequently, digital twin was redefined as a digital replication of living or non-living physical entities, ushering in applications in areas like health and well-being (8). Functioning as a dynamic concept, digital twin embodies a virtual replica of human organs, tissues, cells, or micro-environments that continually adapts to real-time data variations and predicts corresponding future scenarios (9). However, digital twin transcends being a mere digital model linked to its real-life counterpart through emerging technologies. It emerges as a sentient, intelligent, and evolving model, capable of optimizing processes and continuously forecasting future states, such as defects, damages, and failures, through a closed-loop interaction between the digital twin and its surrounding environment.

Broadly, the technologies essential for digital twin can be categorized into two groups: one involves a data-driven statistical model, while the other integrates multi-scale knowledge and data into a mechanical model (10, 11). The numerical model computes structural performance, while the analytical model facilitates structural analysis. An artificial intelligence (AI) model, trained with samples and numerical data, derives real-time structural insights from sensor data.

The impact of digital twin is profoundly reshaping industries and has been embraced by major corporations to heighten efficiency and identify issues. This transformative technology is also finding its way into the healthcare sector. Within this context, digital twin can treat patients as virtualized standalone assets applicable to diverse healthcare scenarios (12). This potential holds substantial promise for improving treatment and diagnostics within hospitals and for individual patients.

Within the realm of healthcare, a digital twin embodies a virtual replica of a tangible entity or process, such as a patient, their anatomical structure, or the setting of a hospital. At present, digital twins in healthcare are designed to dynamically mirror various data sources, including EHRs, disease registries, “-omics” data (such as genomics, biomics, proteomics, or metabolomics data), as well as physical indicators, demographic information, and lifestyle data pertaining to an individual's progression over time (6, 13). The evolution of foundational technologies like the IoT and AI, coupled with the availability of a growing array of accurate and accessible data types (ranging from biometric and behavioral data to emotional, cognitive, and psychological insights), has sparked heightened interest and exploration in the research and potential applications of digital twins within the healthcare domain (13).

### Enhanced patient care through digital twin technology

Digital twin technology has the potential to significantly enhance patient care by leveraging real-time data integration, advanced analytics, and personalized insights (1). These tools can enable healthcare providers to gather and analyze a wealth of patient data from various sources, such as electronic health records (EHRs), medical devices, wearables, and genetic information (2, 14). By integrating and analyzing this data, digital twins create a holistic view of the patient, allowing healthcare professionals to develop personalized treatment plans (6, 9, 15). This approach considers individual patient characteristics, medical history, genetic factors, and real-time physiological data to tailor interventions and medications specifically to the patient's needs, resulting in improved treatment outcomes and patient satisfaction (16–18).

Healthcare professionals could receive support from digital twins in achieving precise and timely diagnoses (19). By analyzing patient data and symptoms, digital twins can simulate various diagnostic scenarios, assisting in differential diagnoses and identifying patterns that may be missed through traditional diagnostic methods alone (14, 20). This improves diagnostic accuracy, reduces errors, and enables earlier intervention, leading to more effective and targeted treatments.

The integration of digital twins with real-time data from wearable devices, remote monitoring systems, and IoT devices enables the ongoing monitoring of patients (21). By monitoring vital signs, physiological parameters, and other health-related data in real-time, digital twins can detect early signs of deterioration or anomalies. This enables healthcare providers to intervene proactively, prevent complications, and optimize treatment plans. Real-time monitoring through digital twins is particularly beneficial for patients with chronic conditions, enabling remote patient management and reducing the need for frequent hospital visits (15, 22, 23).

Digital twins empower patients to actively participate in their own care (24). By providing patients with access to their digital twin data, including personalized health insights, treatment plans, and progress tracking, patients can become more engaged in managing their health (25). This increased engagement leads to better adherence to treatment regimens, lifestyle modifications, and self-management practices. Moreover, digital twins can facilitate communication and collaboration between patients and healthcare providers, promoting shared decision-making and patient-centered care (26).

These tools can leverage predictive analytics and machine learning algorithms to forecast disease progression and treatment outcomes (27). By analyzing patient data and historical trends, digital twins can identify high-risk individuals, predict potential complications, and recommend preventive measures. This proactive approach to care allows healthcare providers to intervene early, prevent adverse events, and optimize treatment plans based on predicted patient responses, ultimately improving patient safety and long-term outcomes (28).

Through secure sharing of patient data across diverse healthcare providers and settings, digital twins can streamline the continuity of care seamlessly (23). This ensures that all involved healthcare professionals have access to the most up-to-date and comprehensive patient information, enabling coordinated care, minimizing duplication of tests, and reducing medical errors. Digital twins could promote efficient and effective communication and collaboration between healthcare teams, enhancing the overall patient care experience (26, 29).

### Predictive analytics and preventive interventions through digital twin technology

Digital twin technology, with its ability to integrate real-time data and advanced analytics, holds great potential for predictive analytics and preventive interventions in healthcare. By leveraging patient data, machine learning algorithms, and predictive modeling, digital twins can identify potential health risks, predict disease progression, and enable proactive interventions (6).

Integrating and analyzing an extensive range of patient data, including medical history, lifestyle factors, genetic information, and real-time physiological data, fosters a comprehensive view of individual health (22). By applying advanced analytics and machine learning algorithms, digital twins can identify patterns, correlations, and anomalies within the data. This could allow healthcare professionals to detect early signs of health risks, such as the development of chronic conditions, adverse reactions to medications, or potential complications. Early detection enables timely interventions and preventive measures to mitigate or manage these risks effectively (30).

Digital twins can simulate disease progression based on patient data and historical trends (9). By analyzing patterns, treatment outcomes, and patient characteristics, digital twins can generate predictive models to forecast disease progression. This information enables healthcare providers to anticipate potential complications, adjust treatment plans, and optimize interventions to slow down or prevent disease progression. Digital twins allow for personalized disease modeling, considering individual patient factors, genetics, lifestyle, and response patterns, leading to more accurate predictions and tailored interventions (31).

Risk stratification can be supported by digital twins, as they categorize patients into varying risk groups according to their health data and predictive models (32). By identifying high-risk individuals, healthcare providers can allocate resources more efficiently, focus on preventive interventions, and implement targeted strategies. Digital twins provide insights into which patients are most likely to benefit from preventive measures, early screenings, lifestyle modifications, or specific interventions. This targeted approach improves the allocation of healthcare resources, reduces costs, and enhances patient outcomes (33).

Digital twins can enable proactive interventions and preventive care by alerting healthcare providers to potential health risks in real-time (34). Through continuous monitoring of patient data, digital twins can detect deviations from normal health parameters, identify early warning signs, and trigger timely interventions. This proactive approach allows healthcare providers to intervene before a condition worsens, preventing hospital admissions, reducing healthcare costs, and improving patient outcomes. Preventive care through digital twins includes personalized health recommendations, reminders for screenings, medication adherence support, and lifestyle modifications (32).

Population health management can be enhanced by digital twins, which analyze collective data from large populations. Employing predictive analytics on this data enables healthcare systems to recognize health trends, risk factors, and disease prevalence patterns at the population level. Digital twins enable healthcare providers to design targeted interventions and preventive strategies at a population level, such as public health campaigns, vaccination programs, or community-based interventions. This population health approach aims to prevent the onset of diseases, improve health outcomes, and reduce the overall burden on the healthcare system (35, 36).

### Optimization of clinical operations through digital twin technology

Digital twin technology offers significant potential for optimizing clinical operations within healthcare systems. By creating virtual replicas of physical systems and integrating real-time data, digital twins enable healthcare providers to analyze and streamline workflows, enhance resource allocation, and improve operational efficiency (19).

Offering a holistic perspective of the clinical workflow, digital twins enable healthcare providers to assess and enhance processes. Through the integration of data from diverse sources like electronic health records (EHRs), medical devices, and administrative systems, digital twins pinpoint bottlenecks, inefficiencies, and areas with potential for improvement (2, 15). This analysis enables healthcare professionals to streamline workflows, reduce redundant tasks, and enhance the overall efficiency of clinical operations.

Contributing to resource allocation optimization within healthcare systems, digital twins offer insights into patient volumes, demand patterns, and resource utilization by analyzing patient data, historical trends, and real-time information (37). This enables healthcare providers to allocate staff, equipment, and facilities effectively, ensuring optimal utilization and minimizing wait times. Digital twins also facilitate capacity planning, allowing healthcare organizations to anticipate future demands and make informed decisions regarding resource investments and expansions.

By predictive analytics and machine learning algorithms, digital twins could aid in operational decision-making. They analyze data from various sources, encompassing patient flow, staffing levels, and equipment usage, enabling them to anticipate future operational scenarios (5). This enables healthcare providers to make proactive decisions, such as adjusting staffing schedules, optimizing bed allocation, or rescheduling procedures, to optimize resource utilization and improve patient care (5).

Integrating seamlessly with real-time data from IoT devices, wearables, and medical sensors, digital twins enable continuous monitoring of clinical operations. Through the monitoring of key performance indicators, patient flow, and operational metrics, digital twins can swiftly identify deviations from anticipated norms and initiate alerts (38, 39). This allows healthcare providers to address issues promptly, such as equipment malfunctions, staffing shortages, or patient bottlenecks, minimizing disruptions and ensuring smooth operations.

Digital twins can support quality improvement initiatives and enhance patient safety within clinical operations (40). By analyzing data on adverse events, near misses, and process variations, digital twins help identify areas where improvements can be made. Healthcare providers can implement evidence-based practices, standardize workflows, and monitor adherence to protocols through digital twins. This fosters a culture of continuous improvement, reduces errors, and enhances patient safety across clinical operations.

Enabling collaboration and communication among various departments and healthcare professionals, digital twins establish a shared virtual platform. This platform permits real-time data sharing, facilitates collaboration on patient care plans, and streamlines communication processes (37, 41). By providing a shared virtual platform, digital twins enable real-time data sharing, collaboration on patient care plans, and streamlined communication. This improves care coordination, reduces delays, and enhances interdisciplinary teamwork, ultimately leading to more efficient clinical operations and improved patient outcomes.

Digital twins can support continuous monitoring and iterative improvement of clinical operations (2). By continuously collecting and analyzing data, digital twins provide insights into operational performance over time. Healthcare providers can identify trends, assess the impact of process changes, and iteratively refine operations based on real-time feedback. This iterative improvement process enables healthcare organizations to adapt to evolving needs, address inefficiencies, and continuously optimize clinical operations.

### Training and simulation through digital twin technology

Digital twin technology can offer valuable opportunities for training and simulation in the healthcare sector (28). By creating virtual replicas of physical systems, integrated with real-time data and advanced simulations, digital twins provide a safe and realistic environment for healthcare professionals to enhance their skills, practice complex procedures, and improve decision-making. This section explores how digital twins contribute to training and simulation in healthcare (42).

Healthcare professionals, notably surgeons, can use digital twins to rehearse and enhance their surgical skills in a simulated environment (43). By replicating surgical procedures and simulating different scenarios, digital twins allow surgeons to gain hands-on experience, test different techniques, and improve their proficiency without risk to real patients. This immersive training enhances surgical skills, hand-eye coordination, and decision-making abilities, ultimately leading to improved patient outcomes and safety.

A platform for healthcare professionals to simulate various medical procedures can be provided by digital twins (6). From invasive interventions to non-invasive techniques, digital twins can replicate procedures and allow healthcare professionals to practice and refine their techniques. This includes simulations of catheter insertions, intubations, ultrasound-guided procedures, and more. By providing a realistic virtual environment, digital twins help healthcare professionals gain confidence, enhance their procedural skills, and ensure patient safety during actual procedures.

Thus, these tools could be particularly valuable for training healthcare professionals in emergency response situations (44). By simulating critical scenarios, such as cardiac arrests, trauma situations, or mass casualty incidents, digital twins allow healthcare providers to practice their response skills, teamwork, and decision-making under high-stress conditions. This training prepares them to handle emergencies effectively, improve coordination, and optimize patient outcomes in real-life emergency situations.

The simulation of intricate clinical cases, providing healthcare professionals with the opportunity to refine and augment their clinical decision-making abilities can be supported by digital twins (45). By incorporating patient data, medical history, and real-time monitoring information, digital twins present healthcare professionals with realistic cases to analyze, diagnose, and develop treatment plans. This interactive simulation provides a valuable learning experience, enabling healthcare professionals to refine their diagnostic reasoning, consider different treatment options, and make informed decisions in a risk-free environment.

By furnishing a shared virtual platform, digital twins facilitate interprofessional collaboration and communication among healthcare professionals from diverse disciplines, enabling them to collaborate effectively (46). Through digital twins, healthcare professionals can practice interdisciplinary teamwork, communicate effectively, and coordinate patient care. This enhances the understanding of each professional's role, fosters collaboration, and improves patient outcomes by ensuring comprehensive and coordinated care.

A platform for continuous professional development for healthcare professionals can be offered by digital twins' use. By providing access to virtual training modules, case studies, and simulation scenarios, digital twins enable healthcare professionals to stay updated with the latest advancements, learn new techniques, and acquire specialized skills. This self-paced learning and continuous training enhance professional development, promote lifelong learning, and ensure healthcare professionals are well-prepared to deliver high-quality care.

Digital twins can also serve as a valuable tool for research and innovation in healthcare (47). Researchers can use digital twins to conduct experiments, analyze data, and test hypotheses. Thus, these tools enable researchers to simulate different patient populations, treatment interventions, and disease scenarios, providing insights that can drive evidence-based practices and innovation in healthcare.

## Conclusion

In conclusion, digital twin technology holds immense promise for revolutionizing healthcare systems and enhancing patient care. By integrating real-time data, advanced analytics, and virtual simulations, digital twins offer personalized treatment plans, predictive analytics, optimized clinical operations, and immersive training opportunities. The use of digital twins empowers healthcare professionals to make accurate diagnoses, monitor patients in real-time, and intervene proactively to prevent adverse events. It also enables patients to actively participate in their own care and promotes collaborative decision-making between patients and healthcare providers. Moreover, digital twins optimize resource allocation, streamline workflows, and improve operational efficiency within healthcare systems. The potential of digital twin technology in healthcare is vast, and its implementation has the potential to significantly improve patient outcomes, enhance patient safety, and drive innovation in the healthcare industry. However, its successful implementation requires addressing challenges related to data privacy, interoperability, data quality, ethics, resource intensity, integration with workflows, validation, education, scalability, and cultural shifts. As these challenges are navigated, digital twin technology could revolutionize healthcare systems, leading to improved patient outcomes, more efficient operations, and a higher quality of care.
